# Virological Non-Suppression among Newly Diagnosed HIV-Positive Individuals on Dolutegravir-Based Antiretroviral Treatment in Eastern Ethiopia: Follow-Up Study

**DOI:** 10.3390/tropicalmed8080391

**Published:** 2023-07-30

**Authors:** Abdella Gemechu, Adane Mihret, Fekadu Alemu Atire, Abraham Aseffa, Rawleigh Howe, Berhanu Seyoum, Andargachew Mulu

**Affiliations:** 1 School of Medical Laboratory Sciences, College of Health and Medical Sciences, Haramaya University, Harar P.O. Box 235, Ethiopia; 2 Armauer Hansen Research Institute, Addis Ababa P.O. Box 1005, Ethiopia

**Keywords:** virological non-suppression, HIV, predictors, baseline viral load, eastern Ethiopia

## Abstract

There have been limited studies linking baseline factors, including the viral load (VL) test, with virological non-suppression since the introduction of dolutegravir (DTG)-based regimens as first-line antiretroviral treatment (ART) in Ethiopia. This study aimed to identify baseline factors associated with virological non-suppression between October 2020 and July 2022. A follow-up study was conducted in eastern Ethiopia among newly diagnosed people living with HIV (PLHIV). A questionnaire and a checklist were used to collect the data. Five milliliters of venous blood were obtained at baseline and six months to determine the VL. A VL test was performed using the Abbott RealTime HIV-1 assay. To determine predictors of virological non-suppression, bivariate and multivariate logistic regression analyses were used. There were 235 PLHIV enrolled, 70.6% of whom were female, with a mean age of 33.9 years. Of the 161 retained on ART, virological non-suppression was 8.7% at six months. Baseline predictors of virological non-suppression were age ≤ 30 years, a history of substance use, and a VL greater than 4-log10 copies/mL. In this cohort, virological non-suppression was found to be optimal but still lagged slightly behind the third 95%–target. Thus, targeted interventions, the introduction of baseline VL testing to improve treatment outcomes, and fostering the attainment of UNAIDS 95–95–95 targets are recommended. Furthermore, broader research is recommended to explore the reasons for virological non-suppression in the study area.

## 1. Introduction

The human immunodeficiency virus (HIV) is a significant threat to public health. Africa is the most severely affected continent, accounting for more than two-thirds of all PLHIV [1]. In Ethiopia, it was estimated that approximately 610,000 people were living with HIV in 2021 [2]. The Joint United Nations Programme on HIV/AIDS (UNAIDS) and the World Health Organization (WHO) encouraged nations and global partners to adopt a series of ambitious strategic target-setting initiatives aimed at eliminating the HIV/AIDS epidemic and improving patients’ access to ART across the world [3]. One initiative will be to reach the UNAIDS 95–95–95 goal, which states that 95% of people with HIV should know their status, 95% of those diagnosed should receive continuous ART, and 95% of those on ART should have a suppressed viral load by 2030 [4]. In 2021, among people accessing treatment, 92% and 68% of all HIV-positive individuals worldwide had viral suppression [1]. In Ethiopia, by the end of the same year, 84%, 78%, and 75% of PLHIV were aware of their HIV status, were on ART, and had achieved viral suppression, respectively [2].

It is well known that virological non-suppression has become a common public health challenge in several African countries due to various multidimensional factors [5]. The estimated pooled prevalence of virological non-suppression among PLHIV in sub-Saharan Africa is 17.25% [6]. Nevertheless, the degree of virological non-suppression varies across nations and study populations, with 34% in central Tanzania [7] and 9–17% in various parts of Ethiopia—9% in Adama [8], 11% in Jimma [9], 12.2% in Delgi [10], 12.5% in the Amhara region [11], 14.5% in Adigrat [12], and 16.6% in Sekota [13].

The ultimate goal of combination ART (cART) for PLHIV is to achieve long-term virological suppression. However, various factors influence virological suppression, including baseline socio-demographic factors, behavioral, clinical, ART, and immunological profiles, and laboratory characteristics. The male gender, being younger in age, and having poor ART adherence, low CD4 count, opportunistic infections, WHO clinical stage III/IV, a history of substance use, ART regimen type, active co-infection with tuberculosis (TB), not using cotrimoxazole prevention, a lack of awareness of the benefits of viral suppression, ART-induced side effects, pretreatment drug resistance, and a high baseline VL are some of the risk factors reported so far [6,14,15,16,17,18,19,20].

The WHO recommended that low-and-middle-income countries (LMIC) carry out VL monitoring at six months, at twelve months, and then annually for people who are stable on ART [4]. Ethiopia’s 2018 national consolidated HIV prevention, care, and treatment guidelines were also recommended and implemented using this approach [21]. In fact, many studies recommend the use of VL monitoring before and after ART initiation when a VL test is available [22]. The baseline VL test is the most important marker of initial assessment in patients with HIV at entry into care, because it determines the VL level of the patient, provides prognostic information about the probability of the disease progression, and monitors the efficacy of ART [23,24]. Evidence suggests that PLHIV with a high VL at the time of ART initiation have higher odds of treatment failure and early disease progression [20,23].

DTG is an integrase inhibitor that was recently approved. It is available as a once-daily dosing for HIV patients, has a high genetic barrier to resistance, and is safer than non-DTG-based cART [25]. However, there were limited data on baseline VL testing before the initiation of ART in LMICs, including Ethiopia, because of its rigorous laboratory work, long turnaround times, and lack of adequate infrastructure and supplies [26]. Furthermore, since the rollout of DTG-based regimens as first-line ART in Ethiopia, no study has linked baseline factors, including the VL test, with virological non-suppression after six months of follow-up on ART. Additionally, studies on the impact of DTG on viral suppression were lacking in the current study area. Thus, this study aimed to determine non-viral suppression and assess its baseline predictors at six months among newly diagnosed PLHIV receiving DTG-based first-line ART in eastern Ethiopia.

## 2. Materials and Methods

### 2.1. Study Setting and Period

**Figure 1:**
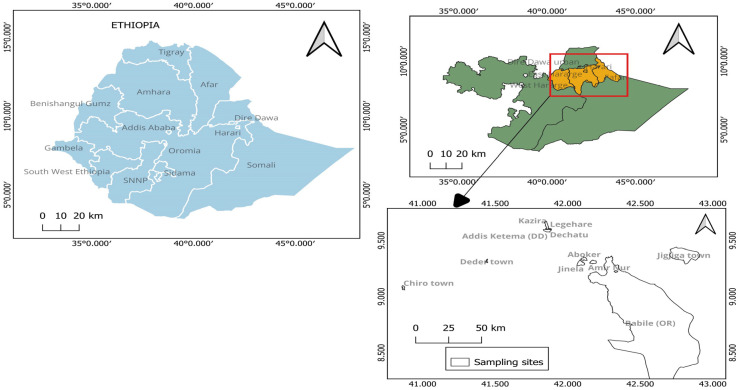
Map of study areas and sampling sites (extracted using QGIS version 3.30.0).

This cohort follow-up study was conducted in 15 selected public health facilities (8 hospitals and 7 health centers) in eastern Ethiopia (Figure 1). The enrollment for the baseline study was conducted between October 2020 and July 2022. The criteria used to select health facilities were the presence of ART care services and the number of PLHIV receiving services at the facility. The health facilities involved were from Harari and Somali Regional State, Dire Dawa City, and the East and West Hararghe zones of the Oromia region. Nearly 80% of PLHIV in the research catchment areas received ART services from the health facilities engaged in this study. In Ethiopia, a comprehensive package for HIV care is provided free of charge. Clinicians and nurses provide clinical care, whereas trained counselors and outreach adherence supporters provide counseling and adherence support. In eastern Ethiopia, a viral load test service is provided at the Harari Health Research and Regional Laboratory and the Dire Dawa Regional Laboratory.

### 2.2. Study Design and Population

A multi-center, health facility-based cohort of PLHIV follow-up study was conducted in eastern Ethiopia. The cohort comprised all newly diagnosed HIV-positive clients who were on DTG-based first-line ART as per the current national guidelines. During the baseline data collection period, study participants were enrolled consecutively and followed up on the ART for the next six months. All the study participants received standard HIV care at participating facilities, including initial ART adherence and routine laboratory monitoring. In addition, available clinical and immunological data at ART initiation were collected. Participants provided blood samples for the baseline VL test at the time of ART initiation and then at six months, as indicated in the national guidelines. Eligible individuals who had already started ART prior to providing a blood sample and those who were critically ill at the enrollment period were excluded from the study.

### 2.3. Sample Size and Sampling Procedure

The sample size was calculated using the WHO sample size determination criterion for HIV pretreatment drug resistance (PDR) [27]. This study was part of a PDR mutation study among newly diagnosed HIV-positive individuals. All study participants enrolled for PDR determination were included upon informed consent and then assessed for their status of virological suppression. At baseline, 235 of 252 (93.25% response rate) newly diagnosed HIV-positive individuals initiating DTG-based first-line ART were included in the cohort. A consecutive sampling method was applied to enroll HIV-positive clients at the selected health facilities, and the proportional distribution of study participants among the facilities was maintained based on the number of parents.

### 2.4. Data and Sample Collection

At baseline, a structured interviewer-administered questionnaire and checklists were used to collect socio-demographic factors, clinical data, laboratory parameters, and information regarding the initiated first-line ART regimen. Study participants or caregivers/guardians of children/adolescents aged less than 18 years were interviewed by trained nurses using a face-to-face interview technique. Moreover, following standard operational procedures, approximately 5 mL venous whole blood samples were collected from all newly diagnosed HIV-positive individuals using tubes containing the anticoagulant ethylene diamine tetra-acetic acid. Plasma samples were harvested by centrifugation of whole blood at 3500 rpm for 5 min and aliquoted into Cryo tubes of 1.8 mL volume. Plasma samples were labelled with the medical registration number (MRN), before the format with some basic information to identify samples was sent to Harari Health Research and Regional Laboratory, Harar, Ethiopia, for HIV-1 RNA VL testing. The samples were then stored at −80 °C for long-term analysis. A similar approach to the baseline data collection method was used at the six-month follow-up. The interviews were conducted, data were collected, and relevant parameters were recorded using checklists. The VL was determined at six months as part of the routine follow-up.

### 2.5. Plasma Viral Load Determination

The collected plasma samples were processed and the VL tests were performed at the Harari Health Research and Regional Laboratory in Harar, Ethiopia. The samples were thawed at room temperature before the VL was run. The Abbott m2000sp automated sample preparation system and the Abbott m2000rt with quantitative Abbott RealTime HIV-1 assay (RT-PCR) (Abbott Molecular Inc., Des Plaines, IL, USA) were used for the extraction of HIV-1 RNA and the determination of plasma VL, respectively. The sample preparation (m2000sp) instrument was used for automated extraction, purification, and preparation of HIV-1 RNA. The m2000rt amplifies, detects, and measures the HIV-1 RNA load. A volume of 0.2 mL of the plasma samples was used for RNA extraction according to the manufacturer’s instructions. The extracted HIV-1 RNA (eluent) was amplified and detected on the m2000rt Abbott platform, both at baseline and at six months of follow-up following the manufacturer’s instructions (https://www.abbottmolecular.com accessed on 8 March 2023). The presence of the HIV-1 target sequence is indicated by the fluorescent signal generated using fluorescently labeled oligonucleotide probes on the Abbott m2000rt machine. The test was performed in the presence of positive, negative, and internal controls. The detection limit of the assay for the plasma sample was over the range of 40–10,000,000 copies/mL.

### 2.6. Data Analysis 

EpiData Manager version 4.6.0.4 was used to code and enter the data. STATA/SE version 14.0 was used for the statistical analysis. For analysis, the log10 transformation of the HIV-RNA VL was used. Demographic and clinical characteristics were summarized using a descriptive analysis. Bivariate and multivariate logistic regression models were used to determine baseline factors associated with virological non-suppression at six months. All variables with *p*-value ≤ 0.25 from bivariate analyses were taken into the multivariable logistic regression analysis to investigate factors independently associated with virological non-suppression. A confidence interval (CI) of 95% and a *p*-value of <0.05 were considered statistically significant.

## 3. Results

### 3.1. Socio-Demographics and Related Characteristics 

In this study, 235 newly diagnosed PLHIV who initiated first-line ART were enrolled, with 70.6% of them female. The mean age of the participants was 33.9 years (SD: ±12.1), with a range of 2–70 years. The overall history of substance use at baseline was 35.3%, and the khat chewing habitual was 34% (80/235) (Table 1).

### 3.2. Baseline Clinical, Laboratory, and ART Profiles

Three-quarters of the study participants were from hospitals (74.9%). More than half of the newly diagnosed participants (60.4%) were linked to ART care through outpatient department (OPD) services. The current TB co-infection was 17%, whereas the overall comorbidity rate at the start of ART was 39.2% (Table 2).

Approximately 72.8% started ART treatment on the same day as diagnosis. At enrollment, 55.7%, 79.6%, 76.2%, 96.2%, and 94.9% of the participants were at WHO clinical stage I, working in functionality status, had a detectable VL (>150 copies/mL), had initiated TDF + 3TC + DTG (1J) first-line ART, and 2NRTI + INSTI classes, respectively. The proportion of participants with baseline HIV RNA VL results greater than 100,000 copies/mL was 23.4%, with results ranging from the target not detected to 4,102,070 copies/mL (Table 2).

### 3.3. Treatment Outcomes and Virological Suppression Status at Six Months 

**Figure 2:**
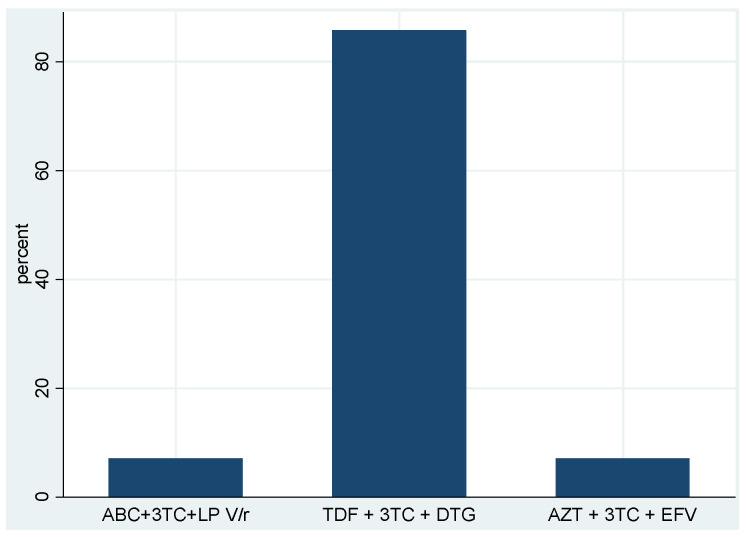
Baseline first-line ART regimen initiated among newly diagnosed HIV-positive individuals with virological non-suppression at 6 months.

The overall ART treatment outcomes at six months were 68.5% retention in ART, 16.6% loss to follow-up (LTFU), 8.9% transferred out, and 5.9% deceased (Table 3). Of the 161 patients retained on ART and available for the analysis, 8.7% (95% CI: 5.2–14.2) failed to achieve virological suppression at six months (Table 3). The median HIV RNA VL among cases with virological non-suppression was 23,301 copies/mL (range: 3252–337,836 copies/mL). The TDF + 3TC + DTG first-line ART regimen was initiated in approximately 85.7% (12/14) of newly diagnosed HIV-positive individuals with virological non-suppression at six months (Figure 2).

### 3.4. Baseline Factors Associated with Virological Non-Suppression at Six Months

Bivariate analysis showed that age ≤ 30 years, WHO clinical stage II and above, a history of substance use, and an HIV RNA VL greater than 4-log10 copies/mL were baseline factors associated with virological non-suppression at six months (Table 4). There was a statistically significant difference in baseline mean VL for PLHIV-achieved viral suppression and non-suppression at six months (140,717.7 vs. 628,569.6 copies/mL; t = −3.0070, *p* = 0.0032). To control for confounding factors, variables with *p*-values of 0.25 in bivariate analysis were imported into a multivariate logistic regression model (Table 4).

Multivariate logistic regression analysis revealed that age ≤ 30 years at ART initiation (AOR = 8.90, 95% CI: 1.85, 42.77), a history of substance use at baseline (AOR = 7.50, 95% CI: 1.41, 39.97), and a baseline HIV RNA VL greater than 4log10 copies/mL (AOR = 12.64, 95% CI: 1.65, 96.48) were independently associated with virological non-suppression at six months. The odds of virological non-suppression at six months were approximately nine times (AOR = 8.90) higher in those younger than 30 years old compared to those older than 30 years old. Moreover, among PLHIV with a history of substance use and a VL greater than 4-log10 copies/mL at baseline, the likelihood of viral non-suppression at six months was 7.5 (AOR = 7.50) and 12.6 (AOR = 12.64) folds higher, respectively (Table 4).

## 4. Discussion

The magnitude of virological non-suppression in this study was 8.7%, and the predictors of virological non-suppression at six months were age less than 30 years, a history of substance use, and a baseline VL > 4-log10 copies/mL. Virological suppression is an important factor in PLHIV health maintenance and plays a great role in the prevention of new HIV cases. In contrast, non-suppression of virological treatment is a key challenge for HIV programs, particularly in LMICs, because the likelihood of drug resistance and subsequent viral transmission, especially that of drug-resistant strains, increases when virological suppression is not achieved.

The rate of virological non-suppression was comparable with studies conducted in various places in Ethiopia: 8.3% at TASH [28], 10.24% in the North Shoa Zone [29], 11.8% in Gondar [30], 12.2% at Delgi Hospital [10], 12% in southern Ethiopia [31], and 10.5% in a study conducted in Dar Es Salaam, Tanzania [32]. Virological non-suppression, however, was lower than that reported from Kenya (24%) [33] and Cameroon (23.2%) [34]. The disparity might be attributed to differences in the study populations, ART follow-up period, advances in ART classes and regimen, study design, the cut-off points to define the virological non-suppression, and adherence [12,25,30]. Additionally, the inconsistency may also be due to the type of interventions and updates in HIV care and treatment guidelines which were periodically optimized to HIV-positive patients toward ART treatments and the type of ARV regimen given up on their routine follow-up [35].

In the current cohort, viral non-suppression was unlikely among PLHIV aged less than 30 years compared to their counterparts. The result was similar with other studies from SSA African countries such as Uganda, Tanzania, and South Africa [36,37,38]. The ART regimen provided to younger HIV-positive people may help explain this. For younger children, a protease inhibitor-based (LPV/r-based) regimen is recommended, though EFV-based regimens can be prescribed. This may affect the virological suppression in the long-term because a high level of drug resistance was already reported in EFV-based regimens [21,39,40]. If the dose is not adjusted with weight increase, the concentration of the drug will be suboptimal, and viral replication cannot be controlled. Weight gain was commonly reported among PLHIV using ART in the dolutegravir era [41,42,43]. Moreover, young individuals have difficulty achieving acceptable ART adherence [38].

This study showed that the likelihood of virological non-suppression was 7.5 folds higher among PLHIV who reported substance use (khat chewing, alcohol drinking, cigarette smoking, etc.) during ART initiation. Studies conducted in South Africa revealed that substance use had higher odds of unsuppressed viral load than those who reported no substance use [44,45]. Moreover, at baseline, stimulant use was associated with inconsistent viral suppression status. This was because substance use was associated with poor adherence. These results highlight the importance of considering substance use in the context of viral suppression [44].

The current study also found that PLHIV with a baseline VL > 4-log10 copies/mL had higher odds of virological non-suppression six months after first-line ART. Previous studies found that a low baseline VL was a predictor of viral suppression (AHR = 1.56) [46], whereas a higher VL at treatment initiation might have a larger HIV reservoir, which might require a longer time to achieve VL suppression than for those with a low VL [47]. Furthermore, studies have shown that HIV-positive individuals with a baseline plasma VL  > 100,000 copies/mL were more likely to fail viral suppression regardless of ARV regimen receiving, including integrase inhibitor-based cART regimens (versus < 100,000 copies/mL) [23,48,49]. In our study, nearly a quarter (23.4%) of the participants had a baseline VL > 100,000 copies/mL during treatment initiation. Therefore, there is an increased risk of HIV transmission to partners when PLHIV carries a higher VL for a longer time after ART initiation. This can provide insights into the interventions that are necessary to enhance health outcomes and mitigate the onward transmission of HIV.

In summary, baseline VL testing is not performed in the majority of LMICs, including Ethiopia, and people who test positive for HIV begin ART without a VL test. In light of this, in addition to the efforts to achieve the targets of the 95s, it is preferable to reconsider the current guidelines for the routine and the first VL test at six months for PLHIV. Not only can baseline VL testing benefit HIV-positive individuals, but it can also assist healthcare professionals in implementing VL-triggered adherence support strategies that may improve patient treatment results [50]. As a result, taking the initiative for baseline VL testing for HIV-positive clients starting ART may be required to properly monitor ART adherence from the start and optimize the benefits of HIV care and treatment programs. Furthermore, cost-effective, affordable, rapid, and decentralized VL testing might increase the uptake of baseline VL and viral suppression in general [26,50,51]. Moreover, considering pretreatment HIV drug resistance genotypic testing at baseline could be helpful in identifying patients at a higher risk of virological failure and contribute to the long-term success of viral suppression [52]. During the inception of ART, focused interventions are also necessary, such as paying attention to younger HIV-positive individuals [38] and those who have substance use habits.

This study has several strengths, including being conducted at multiple sites over a period of six months in eastern Ethiopia. The cohort comprised newly diagnosed PLHIV who started ART in routine HIV care. Baseline factors that can predict virological non-suppression were assessed, including baseline VL testing and the untapped area in Ethiopia. This study provides insight by generating baseline regional epidemiological and clinical data for the health sectors, which will be used for program-wide trend monitoring.

However, this study has some limitations. The result of HIV drug resistance was not included, which could lead to virological non-suppression. The rate of virological non-suppression may be underestimated owing to the relatively small sample size because of the loss to follow-up, transfer out, and deceased participants. Furthermore, those with low baseline viremia at enrollment could deceitfully lower the virological non-suppression at six months. In this study, COVID-19 may have also affected the adherence of the patients. Moreover, immuno-hematological parameters were not adequately captured, as they were not implemented routinely because of their minimal importance and shortage of supplies. Therefore, it is important to consider these limitations when interpreting the conclusions of this study.

## 5. Conclusions

Virological non-suppression after six months of receiving DTG-based first-line ART is found to be optimal in newly diagnosed HIV-positive people. A younger age, a history of substance use, and a VL more than 4-log10 copies/mL at ART inception were baseline predictors of virological non-suppression at six months. Therefore, HIV treatment and care programs, as well as stakeholders, should pay due attention to PLHIV with the identified determinants—potentially through targeted interventions such as optimizing differentiated service delivery for those who did not achieve viral suppression. This finding emphasizes the significance of baseline VL monitoring. To improve treatment outcomes and reduce the incidence of HIV transmission, efforts are needed to introduce cost-effective, rapid, and decentralized baseline VL testing. Furthermore, conducting more comprehensive and broader research is recommended to explore reasons for virological non-suppression in the study area.

## Figures and Tables

**Table 1:** Baseline socio-demographic and behavioral information of PLHIV-initiated DTG-based first-line ART in eastern Ethiopia, 2020/2021 (N = 235).

Variables	Category	Number	Percent (%)
Sex	Male	69	29.4
	Female	166	70.6
Age groups in years	<18	15	6.4
	18–29	71	30.2
	30–39	76	32.3
	40–49	48	20.4
	≥50	25	10.6
Occupational status	Govt employee	33	14.0
	Farmer	11	4.7
	Merchant	34	14.5
	Daily laborer	56	23.8
	Jobless	35	14.9
	Housewife	20	8.5
	Other	31	13.2
	Not applicable *	15	6.4
Marital status	Married	95	40.4
	Single	39	16.6
	Divorced/separated	56	23.8
	Widowed	30	12.8
	Not applicable *	15	6.4
Educational status	No education	83	35.3
	Primary education	68	28.9
	Secondary education	44	18.7
	College or University	25	10.6
	Not applicable *	15	6.4
Other family member with HIV	Yes	100	42.5
	No	135	57.5
History of substance use	Yes	83	35.3
	No	152	64.7
Khat chewing habit	Yes	80	34.0
	No	155	65.9
Alcohol consumption habit	Yes	31	13.2
	No	204	86.8
Smoking habit	Yes	16	6.8
	No	219	93.2

Not applicable *—category is for children/adolescents.

**Table 2:** Baseline clinical, ART, and laboratory characteristics of a cohort of PLHIV-initiated DTG-based first-line ART in eastern Ethiopia, 2020/2021 (N = 235).

Variables	Category	Number	Percent (%)
Type of health facility	Hospital	176	74.9
	Health center	59	25.1
Baseline comorbidity	Yes	92	39.2
	No	143	60.8
Functionality status	Working	187	79.6
	Ambulatory	29	12.3
	Bed-ridden	19	8.1
AIDS-defining illness	Yes	64	27.2
	No	171	72.8
Current TB history	Yes	40	17.0
	No	195	82.9
Baseline INH eligibility *	Yes	170	87.2
	No	25	12.8
Baseline CPT	Yes	148	62.9
	No	87	37.0
Gateways to ART care	VCT/ART clinic	45	19.1
	OPD/MCH	142	60.4
	Family	10	4.3
	Other	38	16.2
Baseline WHO clinical stages	I	131	55.7
	II	33	14.0
	III	51	21.7
	IV	20	8.5
Initiated ART classes	2 NRTI + INSTI	226	96.2
	2 NRTI + PI	3	1.3
	2 NRTI + NNRTI	6	2.5
Baseline first-line ART regimens	TDF + 3TC + DTG	223	94.9
	AZT + 3TC + EFV	4	1.7
	ABC + 3TC + DTG	3	1.3
	ABC + 3TC + LP V/r	3	1.3
	TDF + 3TC + EFV	2	0.9
Number of ART pills/day	One pill/day	231	98.3
	≥2 pills/day	4	1.7
Same-day ART initiation	Yes	171	72.8
	No	64	27.2
Time from diagnosis to ART initiation	Within 7 days	18	7.7
	8–15 days	17	7.2
	>15 days	29	12.3
Baseline CD4 cell count availability	Yes	91	38.7
	No	144	61.3
Baseline hemoglobin availability	Yes	131	55.7
	No	104	44.3
Baseline viral load results (copies/mL)	Target not detected	39	16.6
	<150	17	7.2
	>150	179	76.2
Baseline viral load results category (copies/mL)	≤1000	72	30.6
	1001–10,000	51	21.7
	10,001–100,000	57	24.3
	>100,000	55	23.4
Baseline HIV RNA VL median (range) copies/mL (n = 179)		38,098 (156–4,102,070)	

INH—Isoniazid, CPT—Cotrimoxazole preventive therapy, NRTI—Nucleoside/tide reverse transcriptase inhibitor, NNRTI—Non-nucleoside/tide reverse transcriptase inhibitor, INSTI—Integrase strand transferase inhibitor. * *The denominator is not 235*.

**Table 3:** Treatment outcomes, clinical, ART, and laboratory characteristics of a cohort of PLHIV at 6 months in eastern Ethiopia, 2020/2021 (N = 161).

Variables	Category	Number	Percent (%)
Treatment outcomes	Alive	161	68.5
	Loss to follow-up	39	16.6
	Transferred out	21	8.9
	Died	14	5.9
ART adherence	Good	154	95.7
	Fair/poor	7	4.3
Comorbidities	Yes	16	9.9
	No	145	90.1
Functionality status	Working	154	95.6
	Ambulatory	4	2.5
	Bed-ridden	3	1.9
AIDS-defining illness/events	Yes	10	6.2
	No	151	93.8
WHO clinical stages	T1	151	93.8
	T2	5	3.1
	T3	5	3.1
First-line ART regimen initiated	TDF + 3TC + DTG	156	96.9
	ABC + 3TC + LP V/r	2	1.2
	AZT + 3TC + EFV	1	0.6
	AZT + 3TC + LPV/r	1	0.6
	TDF + 3TC + EFV	1	0.6
History of treatment interruption	Yes	2	1.2
	No	159	98.8
ARV substitution	Yes	1	0.6
	No	160	99.4
Viral load results at 6 months (copies/mL)	Target not detected	131	81.4
	≤150	15	9.3
	151–999	1	0.6
	≥1000	14	8.7
Virological suppression status	Suppressed	147	91.3
	Non-suppressed	14	8.7

**Table 4:** Bivariate and multivariate logistic regression analysis of baseline predictors for virological non-suppression at 6 months among PLHIV cohort in eastern Ethiopia 2020/2021 (N = 161).

Characteristics	Number (%)	Virological Status at 6 Months		Bivariate	Multivariate	
		Non-Suppressed N (%)	Suppressed N (%)	COR (95% CI)	AOR (95% CI)	*p*-Value
Sex						
Male	47 (29.2)	7 (14.9)	40 (85.1)	2.67 (0.88, 8.11)	1.01 (0.21, 4.8)	0.995
Female	114 (70.8)	7 (6.1)	107 (93.9)	1.00	1.00	
Age groups (in years)						
≤30	64 (39.7)	10 (15.6)	54 (84.4)	4.30 (1.3, 14.39)	8.90 (1.85, 42.8)	**0.006**
>30	97 (60.3)	4 (4.1)	93 (95.9)	1.00	1.00	
Health facility type						
Hospital	118 (73.3)	8 (6.8)	110 (93.2)	1.00	1.00	
Health center	43 (26.7)	6 (13.9)	37 (86.1)	2.23 (0.72, 6.84)	4.47 (0.69, 28.8)	0.115
Any comorbidity at baseline						
Yes	64 (39.7)	8 (12.5)	56 (87.5)	2.17 (0.7, 6.57)	2.58 (0.4, 15.3)	0.297
No	97 (60.3)	6 (6.2)	91 (93.8)	1.00	1.00	
Functionality status						
Working	137 (85.1)	12 (8.8)	125 (91.2)	1.00		
Ambulatory/bed-ridden	24 (14.9)	2 (8.3)	22 (91.7)	0.94 (0.19, 4.52)		
Other family members with HIV						
Yes	71 (44.1)	7 (9.9)	64 (90.1)	1.00		
No	90 (55.9)	7 (7.8)	83 (92.2)	0.77 (0.26, 2.31)		
TB history						
Yes	24 (14.9)	2 (8.3)	22 (91.7)	0.95 (0.19, 4.52)		
No	137 (85.1)	12 (8.8)	125 (91.2)	1.00		
INH eligibility						
Yes	125 (91.2)	10 (8.0)	115 (92.0)	1.00		
No	12 (8.8)	2 (16.7)	10 (83.3)	2.3 (0.44, 11.97)		
CPT						
Yes	99 (61.5)	7 (7.1)	92 (92.9)	1.00		
No	62 (38.5)	7 (11.3)	55 (88.7)	1.67 (0.56, 5.02)	2.34 (0.55, 9.9)	0.250
WHO clinical stage						
Stage I	90 (55.9)	4 (4.4)	86 (95.6)	1.00	1.00	
Stage II–IV	71 (44.1)	10 (14.1)	61 (85.9)	3.5 (1.06, 11.76)	2.89 (0.51, 16.26)	0.229
Same-day ART Initiation						
Yes	118 (73.3)	9 (7.6)	109 (92.4)	1.00		
No	43 (26.7)	5 (11.6)	38 (88.4)	1.59 (0.50, 5.05)		
Baseline VL category (copies/mL)						
≤4-log10	84 (52.2)	3 (3.6)	81 (96.4)	1.00		
>4-log10	77 (47.8)	11 (14.3)	66 (85.7)	4.5 (1.21, 16.80)	12.64 (1.65, 96.5)	**0.014**
History of substance use						
Yes	56 (34.8)	9 (16.1)	47 (83.9)	3.8 (1.22, 12.06)	7.50 (1.41, 39.97)	**0.018**
No	105 (65.2)	5 (4.8)	100 (95.2)	1.00	1.00	

INH—Isoniazid, CPT—Cotrimoxazole preventive therapy, VL—Viral load, *p*-values (**bold**)—statistically significant.

## Data Availability

The data presented in this study are available on reasonable request from the corresponding author. The data are not publicly available due to privacy.
